# Cost-effectiveness of roflumilast as an add-on treatment to long-acting bronchodilators in the treatment of COPD associated with chronic bronchitis in the United Kingdom

**DOI:** 10.1007/s10198-013-0456-5

**Published:** 2013-02-08

**Authors:** Yevgeniy Samyshkin, Robert W. Kotchie, Ann-Christin Mörk, Andrew H. Briggs, Eric D. Bateman

**Affiliations:** 1 IMS Health, 210 Pentonville Road, London, N1 9JY UK; 2 IMS Health, 11 Waterview Blvd, Parsippany, NJ 07054 USA; 3 Takeda Pharmaceuticals International GmbH, Thurgauerstrasse 130, 8152 Glattpark-Opfikon, Zurich, Switzerland; 4 Health Economics & Health Technology Assessment, Institute of Health & Wellbeing, University of Glasgow, 1 Lilybank Gardens, Glasgow, G12 8RZ UK; 5 Oxford Outcomes Ltd, Seacourt Tower, West Way, Oxford, OX2 0JJ UK; 6 Department of Medicine, Faculty of Health Sciences, University of Cape Town, George Street, Mowbray, Cape Town, 7700 South Africa

**Keywords:** COPD, Cost-effectiveness analysis, Chronic-obstructive pulmonary disease, Roflumilast, Exacerbations, C, C6, D, I, I1, I19

## Abstract

**Objective:**

To estimate the cost-effectiveness of adding a selective phosphodiesterase-4 inhibitor, roflumilast, to a long-acting bronchodilator therapy (LABA) for the treatment of patients with severe-to-very severe chronic obstructive pulmonary disease (COPD) associated with chronic bronchitis with a history of frequent exacerbations from the UK payer perspective.

**Methods:**

A Markov model was developed to predict the lifetime cost and outcomes [exacerbations rates, life expectancy, and quality-adjusted life years (QALY)] in patients treated with roflumilast, which showed a reduction in the exacerbation rates and lung function improvement in a pooled analysis from two clinical trials, M2-124 and M2-125. Sensitivity analyses were conducted to explore the impact of uncertainties on the cost-effectiveness.

**Results:**

The addition of roflumilast to concomitant LABA reduced the number of exacerbations from 15.6 to 12.7 [2.9 (95 % CI 0.88–4.92) exacerbations avoided] and increased QALYs from 5.45 to 5.61 [0.16 (95 % CI 0.02–0.31) QALYs gained], at an incremental cost of £3,197 (95 % CI £2,135–£4,253). Cost in LABA alone and LABA + roflumilast were £16,161 and £19,358 respectively. The incremental cost-effectiveness ratios in the base case were £19,505 (95 % CI £364–£38,646) per quality-adjusted life-year gained and 18,219 (95 % CI £12,697–£49,135) per life-year gained. Sensitivity analyses suggest that among the main determinants of cost-effectiveness are the reduction of exacerbations and the case fatality rate due to hospital-treated exacerbations. Probabilistic sensitivity analysis suggests that the probability of roflumilast being cost-effective is 82 % at willingness-to-pay £30,000 per QALY.

**Conclusions:**

The addition of roflumilast to LABA in the treatment of patients with severe-to-very severe COPD reduces the rate of exacerbations and can be cost-effective in the UK setting.

## Introduction

Chronic obstructive pulmonary disease (COPD) is a chronic disease characterised by airflow limitation that is only partially reversible and progresses over time. It is a major cause of disability and death worldwide and has an estimated prevalence in Europe of 7.6 %, with the prevalence of physiologically defined COPD in adults ≥40 years of approximately 9–10 % [1]. COPD patients present with symptoms of cough, sputum production, or dyspnoea, and a history of exposure to risk factors, of which smoking is the most important. Symptoms, including activity limitation, and COPD exacerbations place a heavy burden on patients and impair their quality of life [2–4]. Spirometric criteria used to confirm the diagnosis of COPD are a post-bronchodilator FEV_1_/FVC (Tiffeneau index) of <0.7. COPD is judged as severe when the FEV_1_ is between 50 and 30 % and very severe when it is below 30 % of the predicted value [2, 5].

Exacerbations of COPD are characterised by a change in the patient’s baseline dyspnoea, cough, and/or sputum that is beyond normal day-to-day variations, are generally acute in onset, and may prompt a change in regular medication [2]. Exacerbations vary in severity and are responsible for much of the cost of managing COPD. In most clinical and health economics studies of COPD, the severity of an exacerbation is defined by the level of health resources required in their management. Moderate exacerbations are those requiring oral or parenteral corticosteroids [6–8], while severe exacerbations are those leading to hospital admission and/or death.

The frequency of exacerbations are higher in COPD patients with chronic cough [9], and the risk of all-cause mortality is higher in COPD patients with a chronic bronchitis [10, 11]. The impact of exacerbations requiring hospital admission upon health status is particularly severe, affecting mobility and self-care [12, 13].

The goals of management of COPD are to limit disease progression, through reduction of risk factors such as smoking, to reduce the frequency of exacerbations, and to improve quality of life. Pharmacological management involves a stepwise increase in treatment according to a combined assessment of “risk” based on symptoms, severity of lung function impairment, and risk of exacerbations [2], beginning with the as-needed use of short-acting bronchodilators (SABA) followed by the addition of one or more long-acting bronchodilators (LABA) or a long-acting antimuscarinic (LAMA) with or without an inhaled corticosteroid (ICS) [2]. With increasing severity of COPD the use of combinations of these treatments is recommended [5]. None of these treatments have been shown conclusively to modify the long-term decline in lung function that characterises COPD.

In 2010, roflumilast, a selective phosphodiesterase-4 inhibitor with antiinflammatory properties, was approved by European Medicines Agency (EMA) for use as once-daily oral maintenance treatment for adult patients with severe and very severe COPD associated with chronic bronchitis and a history of frequent exacerbations as an add-on to bronchodilators [14]. The effect of roflumilast on exacerbation rates in patients with COPD was investigated in two placebo-controlled double-blind multicentre trials (M2-124 and M2-125) with similar populations of patients with COPD [6]. Both studies compared roflumilast add-on therapy with placebo in patients with a mean age of 64.7 years with a clinical diagnosis of severe-to-very severe COPD and a chronic productive cough and were designed so that approximately 50 % of patients received concomitant treatment with LABA, a pre-specified subgroup of patients [15]. This enabled the comparison of roflumilast plus LABA versus LABA alone. Analysis of the combined patient population in both studies [6] showed a statistically significant reduction of exacerbation rates in patients treated with roflumilast added to concomitant LABA compared to patients treated with LABA alone [relative reduction of exacerbation rates of 0.793 (95 % CI 0.690–0.911)] [15] and a statistically significant increase in post-bronchodilator FEV_1_ of 46 ml (95 % CI 28–64). A further analysis of efficacy confirmed that the difference in exacerbation rates between treatments was independent of concomitant LABA [6] and that the time to first and second exacerbations was lengthened with roflumilast treatment [15].

The objective of the cost-effectiveness analysis reported in this article is to assess the cost-effectiveness of concomitant LABA + roflumilast versus LABA alone, as consistent with the label for roflumilast. The analysis is based on the pooled evidence from trials M2-124 and M2-125 [6, 15, 16] and the current indication for roflumilast.

## Methods

### Model structure

A state-transition Markov model was constructed to estimate the life-time cost and health outcomes and the cost-effectiveness of roflumilast added to concomitant LABA treatment in a cohort based on the patient population in the studies M2-124 and M2-125. Patients’ characteristics in the model cohort, such as mean age, proportion of males, rates of exacerbations in the LABA alone group, the proportion of exacerbations treated in hospital, and the relative reduction of exacerbation rates (RRR) and FEV_1_ improvement in patients treated with roflumilast added to concomitant LABA, were based on the LABA subgroup in studies M2-124 and M2-125 [6, 15] and are presented in Table 1. The model was implemented in the TreeAge Pro 2009 Suite (TreeAge Software Inc.) with a Microsoft^®^ Office Excel 2003 (Microsoft Corporation) front end.

**Table 1:** Base case and key model inputs

Parameter	Value	Distribution in PSA
Age of the cohort at the start of the model, years^a^	64	N/A
Proportion of patients who start the model in severe COPD	1.0	N/A
Mean FEV_1_% in severe COPD at the start of the model, %	40	N/A
Proportion of males in the cohort^a^	0.75	N/A
Mean height of males in the model,^a^ cm	171.3	N/A
Mean height of females in the model,^a^ cm	161.7	N/A
Rate of exacerbations in the severe COPD state in the LABA group,^b^ per patient per year	1.458 (95 % CI 1.292–1.646)	Gamma
Rate of exacerbation in the very severe COPD state in the LABA group,^b^ per patient per year	1.776 (95 % CI 1.447–2.179)	Gamma
Proportion of exacerbations that require hospitalisation in the severe COPD state^c^	0.155 (95 % CI 0.133–0.178)	Beta
Proportion of exacerbations that require hospitalisation in the very severe COPD state	0.244 (95 % CI 0.208–0.280)	Beta
Relative ratio of exacerbation rates in the LABA + roflumilast arm versus LABA alone^d^	0.794 (95 % CI 0.7–0.91)	Lognormal
Post-bronchodilator FEV_1_ improvement due to roflumilast,^e^ L	0.046 (95 % CI 0.028–0.064)	Gamma
Duration of function benefit in the LABA + roflumilast arm, years	1	N/A
FEV_1_ decline per year with COPD,^f^ L	0.052 (95 % CI 0.031–0.062)	Gamma
SMR_B_—for background mortality (excluding hospital death) in the severe COPD state^g^	2.5	Uniform
SMR_B_—for background mortality (excluding hospital death) in the very severe COPD state^g^	3.85	Uniform
Probability of death due to hospital-treated exacerbations (case fatality rate), at age 73^h^	0.077 (SE = 0.044)	Beta
Utility for the severe COPD state^b^	0.751 (95 % CI 0.738–0.765)	Beta
Utility for the very severe COPD state^b^	0.657 (95 % CI 0.635–0.678)	Beta
Annual utility reduction associated with a community-treated exacerbation^i^	0.01 (95 % CI 0.0–0.024)	Beta
Annual utility reduction associated with a hospital-treated exacerbation^i^	0.042 (95 % CI 0.0–0.06)	Beta
Monthly cost of roflumilast,^j^ £	38.23	N/A
Monthly cost of LABA, £^k^	29.67	N/A
Average cost of severe (hospital-treated) exacerbation, £^l^	1,381 (95 % CI 1065–1698)	Gamma
Average cost of community-treated exacerbation, £^m^	66 (95 % CI 41–90)	Gamma
Cost of maintenance of patients in severe COPD, per month, £^n^	48 (95 % CI 34–61)	Gamma
Cost of maintenance of patients in very severe COPD, per month, £^n^	149 (95 % CI 122–176)	Gamma

^a^Based on [6]

^b^Derived from pooled analysis of clinical studies M2-124 and M2-125 [16]; reported in [38]

^c^Reported in [19]

^d^Reported in [15] and adjusted to eliminate the potential double-counting due to indirect reduction of exacerbation rates resulting from the lung function benefit

^e^Reported in [38]

^f^Lung function decline in [18]

^g^Based on [22], estimated for the UK population in NICE COPD Guideline CG101[5]

^h^Based on [20] and adjusted to age of patients in modelled cohort

^i^In the model, the annual reduction of utilities due to an event of exacerbation reported in [23] was accounted for a 1-month model cycle

^j^Roflumilast: estimated manufactured price at the recommended daily dose of 500 μg

^k^LABA: Serevent^®^, at daily dose of 50 μg [24]

^l^Non-elective HRG, including short-stay admissions [25]

^m^Includes cost of a GP visit, cost of an accident and emergency admission, and medications

^n^Based on resource use reported in [12] and respective UK unit costs

**Fig. 1:**
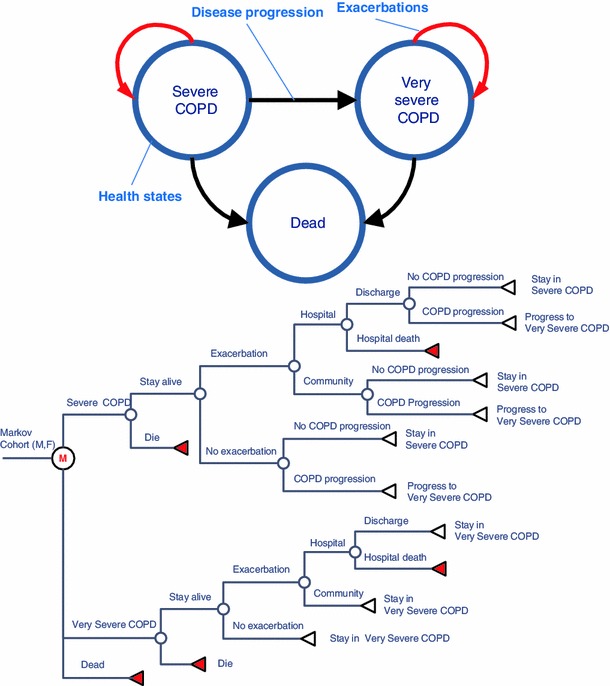
COPD Model structure states and transitions. Transition from severe COPD to very severe COPD is irreversible. In the *tree diagram*, the model cohort is represented by males and females separately because of the differences in rates of the disease progression and background death for males and females. *Encircled M* Markov node, *circle* chance node, *left pointing triangle* terminal node (transition to a different state or exacerbation within the current state)

The model included three states: severe COPD, very severe COPD, and dead. COPD states in the model are based on the severity of the disease as defined by the GOLD criteria [2], i.e. using post-bronchodilator FEV_1_% predicted value relative to the normal population. The model cycle length is 1 month, and the time horizon of the model is 30 years, which is considered a lifetime horizon, given the mean age of patients at start of 64.7 years. Transitions in the model simulate the progression of COPD, exacerbations, and death. Exacerbations are modelled as events that can occur in the severe and very severe COPD states. Patients who experience exacerbations within a model cycle can remain in their respective COPD state, transition to very severe COPD (patients with severe COPD), die because of background mortality, or die as a result of hospital-treated exacerbations. Patients without exacerbations can die because of background mortality, progress to very severe COPD (for patients in the severe COPD state), or remain in their respective COPD state. The disease states and transitions are shown in Fig. 1.

### COPD progression

**Fig. 2:**
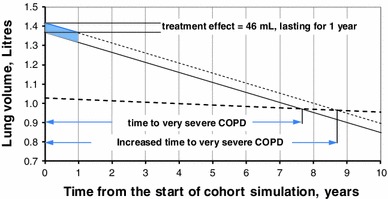
Lung function decline and lung volume improvement (*shaded area* shows the duration of treatment effect for 1 year in the base case). *The diagram* shows the decline of lung volume (FEV_1_) in patients with severe COPD at the rate of 52 ml per year and the 30 % line of lung volume in the general healthy population representing the GOLD definition of very severe COPD. FEV_1_ (males), *L* = (0.0414 × height) − (0.0244 × age) − 2.190; FEV_1_ (females), *L* = (0.0342 × height) − (0.0255 × age) − 1.578 [17. The projected time to progression from the severe to very severe COPD state is estimated at the intercept of the two lines. A lung function benefit is modelled as an increase of lung volume by 46 ml for 1 year and a return to the lung volume of the LABA alone group; the annual rate of decline of lung volume remains the same in the roflumilast + LABA and LABA alone groups

The progression from the severe to very severe COPD state depends on the baseline average FEV_1_ value (L) and the estimated lung volume decline in the COPD cohort. FEV_1_ naturally declines with age in the general population [17], but more rapidly in patients with COPD (52 ml per year [18]). Predictive equations for lung function for males and females in the general population were taken from a study of reference spirometric values in non-smoking men and women [17]: FEV_1_ (men), *L* = (0.0414 × height) − (0.0244 × age) − 2.190; FEV_1_ (women), and *L* = (0.0342 × height) − (0.0255 × age) − 1.578 (height in cm). Monthly probabilities of progression of patients from the severe COPD state to the very severe COPD state were derived from the estimated time required for FEV_1_ in the severe COPD state to reach the 30 % post-bronchodilator FEV_1_% threshold, corresponding to the definition of very severe COPD. The cycle probability of transition from the severe COPD state to the very severe COPD state was estimated as the reciprocal of the average estimated time (*T*) in the severe COPD state for males and females separately. The average estimated time (*T*) in the severe COPD state was derived from the intersection of the line that represents lung function in patients with COPD with the line that represents 30 % of the lung function in the normal population (Fig. 2).

### Exacerbations

Exacerbations in the model are either moderate (i.e. community-treated) or severe (i.e. requiring hospital admission), each associated with costs and health outcomes. Moderate exacerbations in the studies M2-124 and M2-125 were defined as those requiring systemic treatment with oral steroids [6, 15]. The annual rates of exacerbations from a pooled analysis of a pre-specified subgroup of patients treated with concomitant LABA in studies M2-124 and M2-125 [16] were used to estimate the baseline exacerbation rates in the LABA alone arm. A pooled analysis was possible because of the identical design of the studies M2-124 and M2-125 [6]. Exacerbation rates in the treatment arm (LABA + roflumilast) are expressed via the baseline rate of exacerbations in the LABA alone arm and a relative reduction of exacerbation rates (RRR) associated with treatment.

The baseline annual exacerbation rates were estimated separately for patients in the severe and in very severe COPD states and equalled 1.458 (95 % CI 1.292–1.646) and 1.776 (95 % CI 1.447–2.179) respectively. Fixed proportions of all exacerbations in the severe and the very severe COPD states are hospital-treated exacerbations, based on estimates from the pooled analysis of trial data and equalled 0.155 (95 % CI 0.133–0.178) in the severe COPD state and 0.244 (95 % CI 0.208–0.280) in the very severe COPD state [19].

### Mortality

Mortality in the modelled COPD cohort occurs as both a result of case fatality during a hospital-treated exacerbation and as age- and sex-specific background mortality that excludes hospital mortality. In the model, the background mortality and the hospital mortality combined represent the all-cause mortality in populations with severe and very severe COPD.

#### Hospital mortality

The risk of death due to a hospital-treated exacerbation (case fatality rate, CFR) was obtained from the 2008 UK National COPD Audit Report [20] where CFR was reported at 7.7 % for a sample of 9,716 admissions for COPD exacerbations (with subsequently estimated SE of 4.4 %). The average age of patients in the UK COPD Audit Report was 73 years. The average age of patients in the model at the start of the simulation was 64 years. To avoid overestimation of hospital mortality at the start of cohort simulation we assumed that hospital mortality risk would be lower in patients younger than 73 years and increase in patients older than 73 years, and we used the ratio of age-specific risk of death in the general population to the risk of death at the age of 73 to adjust the CFR value of 7.7 %. With such an adjustment, at the age of the cohort of 64 years, the resultant hospital CFR was 3.2 % (where 100 % of the cohort patients were alive), further reaching the value of 7.7 % at the cohort age of 73 years (when 52 % of cohort patients were alive) and approaching the level of 28 % at the age of 85 years (with only 2.4 % of the cohort patients alive), as shown in Table 2. In a scenario analysis, an alternative estimate of the age adjustment of the risk of hospital mortality reported in a meta-analysis study [21] was tested.

**Table 2:** Hospital case fatality rate (CFR) adjusted to age of cohort

Age (M/F), years	64	70	73	75	80	85
Adjustment^a^	0.46	0.73	1.00	1.24	2.15	3.68
Hospital CFR	0.035	0.057	0.077^b^	0.095	0.166	0.283
Proportion of cohort alive (%)^c^	100	69	52	39	15	2.4

^a^Adjustment using the ratio of the age-specific risk of death in the UK general population to the risk of death in the UK general population at the age of 73, the average age in the UK COPD Audit Report [20]

^b^ *Source*: 2008 UK COPD Audit Report [20]

^c^Estimated from model simulation

#### Background mortality and SMR

In the model, the risk of background mortality in the severe and very severe COPD states is expressed via the age- and sex-specific mortality in the general population multiplied by standardised mortality ratios for background mortality that excludes hospital deaths (SMR_B_). The relative mortality risk ratio by COPD GOLD stage versus non-COPD population was based on a Swedish study [22] that provided estimates of the relative COPD mortality risk of the FEV_1_ group compared with the general population mortality. The all-cause mortality SMR in severe COPD was estimated at 3.1 (95 % CI 2.6–4.1) and in very severe COPD 5.0 (95 % CI 3.5–11.8) as the weighted average for smokers, former smokers, and never smokers stratified by GOLD COPD severity stage and gender compared to the general population without symptoms of chronic bronchitis and with normal pulmonary function. The values of SMR_B_ (which exclude hospital deaths) were subsequently estimated by separating the hospital case fatality rate multiplied by the risk of hospital-treated exacerbations from the risk of all-cause mortality in severe and very severe COPD separately and were estimated at 2.5 and 3.85 for the severe and very severe COPD states respectively (shown in Table 3). The impact of the estimated SMR_B_ on the incremental cost-effectiveness was explored in a sensitivity analysis and was found to have little impact on the incremental cost-effectiveness (Table 6).

**Table 3:** Standardised mortality ratios for background mortality by COPD severity stage

	Severe COPD	Very severe COPD
Estimated SMR_B_ for background death^a^	2.50	3.85
All-cause SMR^b^	3.1 (95 % CI 2.6–4.1)	5.0 (95 % CI 3.5–11.8)

^a^Mortality risk by GOLD stage versus non-COPD population (applied to age-specific mortality rates for the UK general population). Derived based on all-cause SMR in [22] and [5]; the mortality risk associated with hospital-treated exacerbations was deducted from all-cause SMR

^b^ *Source*: A Swedish study [22] where SMRs were estimated as weighted average for smokers, former smokers, and never smokers stratified by GOLD COPD severity stage and gender compared to the general population without symptoms of chronic bronchitis and with normal pulmonary function

### Health-related utilities

Two types of health-related utilities are employed in the model. The severe COPD and very severe COPD states are associated with the utility values of 0.751 (95 % CI 0.738–0.765) and 0.657 (95 % CI 0.635–0.678) respectively [16]. Hospital-treated exacerbations and community-treated exacerbations cause loss of utility that is translated in the model into loss of QALY per an event of exacerbation. The QALY loss due to an exacerbation was estimated based on the annual utility reduction of 0.01 (SE = 0.007) and 0.042 (SE = 0.009) associated with a community-treated exacerbation and a hospital-treated exacerbation respectively, reported in [23]. In the model, the loss of QALY was applied to the model cycle within which an exacerbation occurred; therefore the values of absolute reduction of health-related utilities within 1 month in the model equalled 0.12 (representing a loss of QALY per a moderate exacerbation of 0.01) and 0.504 (representing a loss of QALY per severe exacerbation of 0.042) due to a community-treated exacerbation and due to a hospital-treated exacerbation respectively.

### Resource use and costs

The cost-economic analysis was conducted from the perspective of the UK National Health Service (NHS). Costs and outcomes were discounted at 3.5 % per annum. The following direct health care costs are considered in the model: (1) monthly costs of maintenance of a patient in the severe and very severe COPD states; (2) monthly costs per patient of roflumilast + LABA and LABA, and (3) event costs of community-treated and hospital-treated exacerbations. Maintenance costs included outpatient visits, oxygen therapy, spirometry tests, and influenza vaccinations [12] and amounted to £48 (95 % CI £34–£61) and £149 (95 % CI £122–£176) per month for patients with severe and very severe COPD respectively (Table 4). The monthly costs of roflumilast was £38.23 (estimated manufacturer price at the recommended daily dose of 500 μg), and the monthly cost of LABA was £29.67 [Serevent^®^ (Salmeterol^®^)] at a daily dose of 50 μg [24]. The cost of a community-treated exacerbation was estimated at £65 (95 % CI £41–£89). The cost of a hospital-treated exacerbation was based on non-elective Healthcare Resource Groups (HRGs) weighted by volume [25], related to COPD, that also included HRGs for short hospital stays and was estimated at £1,381 (95 % CI £1,065–£1,698) (Table 4).

**Table 4:** Cost inputs

Cost category	Unit cost, £	Resource use	Proportion of patients	Cost, £	Reference
*Community-treated exacerbation*					
GP visit	35	1	2/3	23.33	GP cost per 11.7-min consultation, PSSRU [39]
Accident & Emergency (not leading to admission)	106	1	2/3	35.33	NHS Reference Costs, Accident and Emergency Services: not leading to admission
Prednisolone 30 mg	0.58	7 days	1/2	2.03	Assumed 1 × 2 5 mg tablet and 1 × 5 mg tablet. This isn’t the cheapest option; however it is the combination that produces the lowest pill burden. Taken for 7–14 days (NICE 2010 [5]). 50 % given for 7 days, and 50 % given for 14 days [24]
Prednisolone 30 mg	0.58	14 days	1/2	4.05	
Cost of a community-treated exacerbation, £				64.74	
*Hospital-treated exacerbation*					
Hospital admission for COPD	1193.41		1	1,193.41	Weighted average of HRG, non-elective long and short stay [25] (HRG DZ21A to DZ21 K)
Ambulance transport	208.95		0.9	188.06	Paramedic services: category B/amber [incidents of category B (amber) calls, defined as ‘patients who require urgent face-to-face clinical attention but are not immediately life threatened’. PSO6B: 06 breathing problems; breathing difficulty. Assume 94 % require ambulance transport [25]
Cost of a hospital of treated exacerbation				1,381.47	

Cost category	Unit cost, £	Resource use	Cost, £	Reference
*Maintenance of patients with severe COPD*				
Outpatient visit to respiratory physician	131.74	2 per year	21.96	UK national reference costs. Consultant led: follow-up. Attendance non-admitted face to face (service code 340) [25]
Spirometry	45.59	2 per year	7.60	Direct access: diagnostic services (spirometry test and bronchodilator response test, currency code DA07) [25]
Influenza vaccination, annual	4.15 per vaccination	75 % patients	0.26	British national formulary [24]
Oxygen therapy	14.72 per day	14.6 days per year = 1.22 days monthly	17.91	
Cost of maintenance of patients with severe COPD, £ (monthly), £			47.72	
*Maintenance of patients with very severe COPD*				
Outpatient visit to respiratory physician	131.74	4 per year	43.91	
Spirometry	45.59	4 per year	15.20	Direct access: diagnostic services (spirometry test and bronchodilator response test, currency code DA07) [25]
Influenza vaccination, annual	4.15 per vaccination	75 % patients	0.26	British national formulary [24]
Oxygen therapy	14.72 per day	73 days per year 6.08 days monthly	89.54	Price per day reported in [12] converted to GBP at exchange rate
Cost of maintenance of patients with very severe COPD (monthly), £			148.91	

### Reduction of exacerbations

A relative rate reduction (RRR) of exacerbations of 0.793 (95 % CI 0.690–0.911) was derived for severe COPD and very severe COPD combined, using pooled data from trials M2-124 and M2-125 [15] for the pre-specified subgroup of patients treated with concomitant LABA. In the model, this RRR was adjusted to eliminate the potential double-counting due to indirect reduction of exacerbation rates resulting from the lung function improvement (i.e. slowing down the progression of disease to very severe COPD). The adjusted relative reduction of the exacerbation rate ratio applied in the model was 0.794 (95 % CI 0.70–0.91). The reduction of exacerbations due to treatment reduces the effect of exacerbations on the health-related utility and subsequently leads to a reduction in mortality due to hospital-treated exacerbations. In the model it was assumed that the reduction of exacerbations lasts for the lifetime horizon. In one-way sensitivity analysis, shorter durations of the reduction of exacerbations were tested.

### Lung function improvement

Roflumilast add-on treatment has been shown to improve lung function [6]. This treatment effect was implemented in the model as an increase in FEV_1_ (an improvement in post-bronchodilator FEV_1_ of 46 ml (95 % CI 28–64) in patients treated with concomitant LABA + roflumilast. The lung volume increase did not have an impact on the annual decline of lung volume, which remained equal to 52 ml in patients in the treatment and comparator arms. In the base case analysis, the lung function benefit of roflumilast was limited to 1 year, as observed in studies M2-124 and M2-125. After 1 year, it was assumed that the lung volumes in patients in the roflumilast and comparator arms were equal. Improvement of lung function results in an increased time that patients stay in the severe COPD state, delaying the transition to the very severe COPD state. Figure 2 shows the impact of the improvement of lung function on the average estimated time in the severe COPD state. The impact of a longer duration of lung function benefit was explored in a sensitivity analysis.

### Adverse events

The majority of adverse events in patients treated with roflumilast in studies M2-124 and M2-125 were mild-to-moderate and transient and occurred within the first month from the beginning of treatment. In the model base case analysis, adverse events were therefore not taken into consideration. In a sensitivity analysis, an impact of adverse events was assessed by assuming within the first cycle health impact and costs of treatment of adverse events per patient at 50 % of the average cost and 50 % of the health impact of a community-treated exacerbation.

### Probabilistic sensitivity analysis

**Fig. 3:**
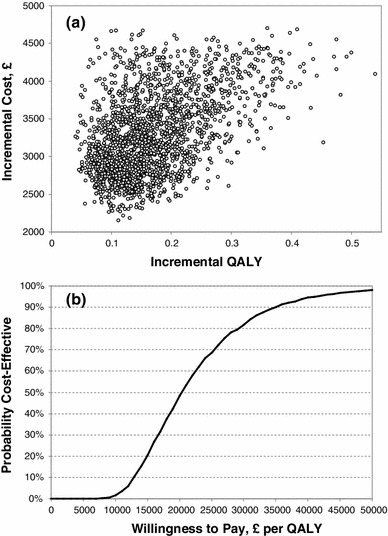
Probabilistic sensitivity analysis: incremental cost-effectiveness scatter plot (**a**) and cost-effectiveness acceptability curve (**b**)

In order to explore the combined impact of all uncertainties in the model inputs on the cost-effectiveness, a probabilistic sensitivity analysis (PSA) was conducted. The list of the input parameters included in the PSA and the corresponding distributions with their respective 95 % CIs are shown in Table 1. The gamma distribution was used for the costs, the annual lung volume decline, the lung volume improvement, and the background rates of exacerbations in the LABA only arm. The beta distribution was used for the proportion of hospital-treated exacerbations, the hospital case fatality rate, and the health-related utilities associated with COPD and the disutilities of exacerbations. Log-normal distribution was applied to the treatment effect (reduction of exacerbation rates). For standardised mortality ratios for background death uniform distributions were used, cantered around the mean values. The number of iterations in the PSA was 2,000; the PSA results are presented in Fig. 3 as an incremental scatter plot and the cost-effectiveness acceptability curve (CEAC).

### A summary of health benefits of roflumilast

The benefits of adding roflumilast to concomitant bronchodilator therapy (LABA) predicted by the model result from the reduction of exacerbation rates and from the improvement in lung function. The reduction of exacerbation rates reduces the QALY loss from exacerbation events and reduces mortality because of the reduction of hospital-treated exacerbations. The improvement in lung function delays the progression of patients from the severe COPD to the very severe COPD state, which is associated with lower health-related utility, higher maintenance costs, higher frequency of exacerbations, and higher background mortality (SMR_B_).

## Results

### Base case analysis and PSA

For the LABA alone arm, the model (Table 5) predicts the discounted life expectancy of 8.0 years (95 % CI 6.84–9.15), the mean quality-adjusted life expectancy (QALY) of 5.45 (95 % CI 3.96–6.94), and the number of exacerbations of 15.64 (95 % CI 9.56–21.72). The cost per patient amounts to £16,161 (95 % CI £10,039–£22,013).

**Table 5:** Cost-effectiveness analysis, base case

	LABA	roflumilast + LABA	Incremental
Costs, £	16,161 (95 % CI 10,039–22,013)	19,358 (95 % CI 12,711–26,050)	3,197 (95 % CI 2135–4259)
QALYs	5.451 (95 % CI 3.96–6.94)	5.615 (95 % CI 4.08–7.15)	0.164 (95 % CI 0.02–0.31)
Life years	8.0 (95 % CI 6.84–9.15)	8.17 (95 % CI 5.84–10.50)	0.175 (95 % CI −0.03 to 0.38)
Number of exacerbations	15.64 (95 % CI 9.56–21.72)	12.74 (95 % CI 7.56–17.92)	−2.9 (95 % CI 0.88–4.92)
Cost per QALY gained, £	19,505 (95 % CI 364–38,646)		
Cost per LY gained, £	18,219 (95 % CI −12,697 to 49,135)		

For the LABA + roflumilast arm, the mean discounted life expectancy yields 8.17 years (95 % CI 5.84–10.50), quality-adjusted life expectancy 5.61 (95 % CI 4.08–7.15) QALYs, and 12.7 exacerbation (95 % CI 7.56–17.92). Treatment with add-on roflumilast resulted in incremental life expectancy 0.18 years (95 % CI −0.03 to 0.38), incremental QALYs 0.16 (95 % CI 0.02–0.31), and 2.9 fewer exacerbations (95 % CI 0.88–4.92). The incremental cost-effectiveness ratio (ICER) associated with adding roflumilast to LABA amounts to £19,505 (95 % CI £364–£38,646) per QALY gained and to £18,219 per life-year gained (95 % CI −£12,697 to £49,135).

### Probabilistic sensitivity analysis (PSA)

The uncertainty in the cost-effectiveness was explored in a probabilistic sensitivity analysis using Monte-Carlo simulation [26]. The cost-effectiveness scatter plot and cost-effectiveness acceptability curve (CEAC) are presented in Fig. 3; the probability of roflumilast as an add-on to LABA versus LABA alone being cost-effective is 82 % at willingness-to-pay £30,000 per QALY.

### One-way sensitivity analysis (OWSA)

A range of one-way sensitivity analyses (OWSA) were performed to explore the impact of uncertainty in model parameters on the incremental cost-effectiveness. The lower and upper estimates for variables in the OWSA were based, where possible, on the boundaries of the respective 95 % confidence intervals, or on the assumptions, and are presented in Table 6.

**Table 6:** Uncertainty in model parameters and one-way sensitivity analysis

Sensitivity and scenario analyses	Parameter values				ICER, £/QALY	
	Base case values (95 % CI)	Low estimate	High estimate	Source	At parameter low estimate	At parameter high estimate
Base case incremental cost-effectiveness ratio, £ per QALY gained					19,505	
Discount rate, costs (% per annum)	3.5 %	0 %	6 %	Assumption	24,377	17,001
Discount rate, effects (% per annum)	3.5 %	0 %	6 %	Assumption	14,111	23,919
Discount rate costs and effect (% per annum)	3.5 %	0 %	6 %	Assumption	17,636	20,848
Cohort characteristics—proportion of males (males:females)	0.75:0.25	0:1.0	1.0:0	Assumption	17,153	20,587
Proportion of patients in cohort who start in severe COPD	0 (all start in very severe COPD)	1.0 (all start in severe COPD)	1.0	Assumption	17,792	19,505
Mean cohort age at start of model (years)	64	60	70	Assumption	20,824	17,752
Relative ratio of exacerbation rates	0.794	0.70	0.93	95 % CI	12,829	53,435
Rate of exacerbations in the severe COPD state in the LABA group (per patient per year)	1.458	1.292	1.646	95 % CI	20,475	18,501
Rate of exacerbations in the very severe COPD state in the LABA group (per patient per year)	1.776	1.447	2.179	95 % CI	21,876	17,217
Proportion of exacerbations that require hospitalisation in the severe COPD state	0.155	0.133	0.178	95 % CI	20,439	18,608
Proportion of exacerbations that require hospitalisation in the very severe COPD state	0.244	0.208	0.280	95 % CI	21,005	18,207
Utility in severe COPD	0.751	0.738	0.765	95 % CI	19,671	19,329
Utility in very severe COPD	0.657	0.635	0.678	95 % CI	19,687	19,334
Utility decrement for a community-treated exacerbation (absolute, applied for 1 month)	0.12	0.00	0.29	95 % CI	22,117	16,707
Utility decrement for a hospital-treated exacerbation	0.504	0.21	0.72	95 % CI	20,908	18,569
Hospital mortality—CFR at age 73	0.077	0.0	0.14	Assumption	47,963	15,031
SMR_B_ (excluding hospital mortality) in severe COPD	2.5	1 (no increase of mortality)	3.85 (same as in very severe COPD)	Assumption	18,183	20,534
SMR (excluding hospital mortality) in very severe COPD	3.85	2.5 (same in severe COPD)	5.2	Assumption	18,495	20,419
SMR_B_ (excluding hospital mortality) in the severe and very severe COPD states	2.50 and 3.85 respectively	Base case:50 % for both SMR_B_	Base case + 50 % for both SMR_B_	Assumption	16,363	21,777
Annual lung volume decline in COPD population, litres	0.052 (95 % CI 0.031–0.062)	0.031	0.062	95 % CI	20,844	19,052
Lung function benefit for roflumilast, litres	0.046 (95 % CI 0.028–0.064)	0.028	0.064	95 % CI	20,312	18,818
Duration of lung function benefit, years	1	No benefit	5	Assumption	21,901	14,889
Cost of severe COPD disease management, £ per month	48	34	61	95 % CI	19,398	19,608
Cost of very severe COPD disease management, £ per month	149	122	176	95 % CI	20,083	20,504
Cost of a community-treated exacerbation, £	65 (95 % CI 41–89)	41	89	95 % CI	19,785	19,218
Cost of a hospital-treated exacerbation, £	1381 (95 % CI 1065–1698)	1065	1698	95 % CI	20,445	18,563
Adverse events	No AE		50 % cost of moderate exacerbation and 50 % of loss of QALY due to moderate exacerbation	Assumption	19,505 (base case)	20,322

### Relative reduction of exacerbation rates

The predicted cost-effectiveness is most sensitive to the relative reduction of exacerbations rates (RRR) and varied from £12,829 per QALY gained to £53,435 per QALY gained when RRR varied from the lower to upper bounds of its 95 % CI respectively. In the model, it is assumed that the treatment effect of reduction of exacerbation rates applies for the entire duration of simulation (a lifetime horizon) as there is no evidence from the roflumilast studies to suggest a reduction of the treatment effect of roflumilast with time. However, a scenario analysis was performed to explore the cost-effectiveness with the assumption of a limited duration of the treatment effect. In that scenario analysis, both the treatment effect and the cost of roflumilast were removed after a number of years. In this scenario analysis the incremental cost-effectiveness ratio (ICER) remained stable within a wide range of assumptions on the duration of treatment effect (years): £10,966 (1 year), £16,515 (2 years), £20,369 (5 years), £20,298 (10 years), and £19,543 (20 years) per QALY gained.

### Hospital CFR

In the absence of hospital mortality, the ICER was estimated at £47,963 per QALY. At higher CFR (14 % at age 73), the ICER was £15,031 per QALY gained. This upper limit of 95 % CI of hospital CFR at age 73 in the model is close to the CFR value of 15.6 % (95 % CI 10.9–20.3) reported in a meta-analysis study [21].

This age adjustment of hospital CFR in the model results in a lower risk of hospital CFR at the beginning of simulation at age 64 (approximately 3.3 %) compared to the age adjustment to CFR reported in [21] (4 % increase per 1 year of age). In a sensitivity analysis, we applied a CFR of 0.077 at age 74 and adjusted it by increasing the risk of death by 4 % per year of age in patients older than 73 and by reducing the risk of death by 4 % per year in patients younger than 73. This resulted in an ICER of £17,720 per QALY gained. An assumption of constant CFR of 0.077 regardless of cohort age results in an ICER of £16,205 per QALY gained. The assumption taken in the model represents therefore a more conservative approach to estimating the risk of hospital mortality.

### Duration of lung function benefits

At 1-year duration of lung function benefits, the magnitude of improvement of lung function has a moderate impact on the cost-effectiveness: the ICER varied from £20,312 to £18,818 per QALY gained at FEV_1_ benefits ranging from 28 to 64 ml (representing the lower and upper limits of 95 % CI). When no lung function benefit is assumed, the ICER amounts to £21,901 per QALY gained. It such a case, the effect of treatment is determined by the reduction of exacerbations only. An assumption of lung function benefits lasting for 2 and 5 years results in ICERs of £17,712 and £14,889 per QALY gained respectively (Table 6).

### Rate of exacerbations and patients with frequent exacerbations

The cost-effectiveness is more sensitive to the background rate of exacerbations in the very severe COPD state in patients treated with LABA alone [ICER = £17,217 per QALY and £21,876 per QALY gained at the annual exacerbation rates of 2.18 and 1.45 (95 % CI)] than to the rate of exacerbation in the severe COPD state [ICER = £18,501 per QALY and £20,475 at annual exacerbation rates of 1.65 and 1.29 (95 % CI)].

The cost-effectiveness was explored at the annual rate of exacerbations of ≥ 2 per patient in the severe COPD state to represent patients with frequent exacerbations, as defined in the 2011 Global Strategy for the Diagnosis, Management and Prevention of COPD [2]. With the assumed higher annual rates of exacerbations of 2.0, 2.25, and 2.5 in the severe COPD state and the proportionately increased rates of exacerbations in the very severe COPD state, the estimated ICERs yield £14,120, £12,492, and £11,192 per QALY respectively.

### Background mortality

The assumption regarding the values of SMR_B_ for background mortality (excluding hospital mortality) did not have considerable impact on the cost-effectiveness: at SMR_B_ in severe COPD varying from 1.0 to 3.85, the ICER changes from £18,183 to £20,534 per QALY gained respectively. Similarly, varying SMR_B_ in the very severe COPD state from 2.5 to 5.2 changes the ICER from £18,495 to £20,419 per QALY gained.

### Time horizon

The time horizon in the base case was lifetime (30 years in the model). At shorter time horizons the incremental cost-effectiveness was £59,690 per QALY (1 year), £49,824 per QALY (2 years), £44,336 QALY (3 years), £36,909 (5 years), and £26,522 (10 years) per QALY gained.

## Discussion

The economic analysis conducted in this study is built upon the commonly accepted approaches taken in the previously published pharmacoeconomic models in COPD [12, 13, 27–30], with the efficacy of treatment derived from pooled analysis of two clinical trials. Generally, earlier cost-effectiveness studies have modelled COPD with disease states and transitions determined by lung function (FEV_1_) assuming, with some variations, that the effect of treatment leads to the delay of time to transition to more severe disease states and to a reduction of the rates of exacerbations. The existing variety of methods in the application of lung function benefits, mortality, and utilities suggests that there is no consensus on the best practice in the application of these factors in COPD modelling. The accuracy of the model presented in this study and other models is also limited by the evidence from clinical trials that typically have limited duration compared to the natural history of disease and therefore by the need to use external data, such as reduction of health-related utilities due to exacerbations and estimated of background mortality and hospital mortality.

In this study, two key methods were employed to adjust the estimates of hospital mortality and background mortality. First, the model uses the hospital case fatality rate due to severe exacerbations to assess potential survival benefits associated with avoidance of hospital-treated exacerbations, with the hospital case fatality rate sourced from a UK 2008 COPD Audit Report [20]. Subsequently, the value of CFR of 7.7 % reported in the UK COPD Audit Report was adjusted to reflect the difference in age within the modelled cohort (64 years at start) and in patients in the UK COPD Audit Report (73 years). This conservative assumption was made in order not to overestimate deaths from severe exacerbations in younger cohort in the initial period of Markov cohort simulation.

Second, the all-cause SMR generated by the model that comprises SMR_B_ for the background mortality and the hospital CFR was calibrated against all-cause SMR reported in UK Clinical Guideline CG101 [5]. Such separation of SMR_B_ attributed to background mortality and the hospital case fatality rate in the model has enabled greater flexibility regarding ranges of the hospital case fatality rates, making the model applicable to a wider range of settings.

Furthermore, adjustments to the RRR obtained from the studies M2-124 and M2-125 were made to avoid double-counting of the impact of lung function improvement on the reduction of exacerbations. Because the results with roflumilast reported to date are based on 12-month studies, a conservative approach was adopted considering that the lung function benefits would last for 1 year only. However, evidence from longer treatment trials in COPD (3- or 4-year duration) suggests that the benefit of effective treatments persists for years (TORCH [31, 32] and UPLIFT [33, 34]).

The following key assumptions regarding the efficacy of roflumilast were made in the model. The model employs a single pooled ratio of reduction of exacerbation rates (RRR) that has been shown to be statistically significant in two pivotal trials [15] that had identical designs. The single RRR is applied to both the severe and very severe COPD states, as the statistical analysis of trial data demonstrated that the reduction of exacerbation rates was independent of COPD severity. It was also assumed in the model that the relative rate of reduction of exacerbations remains constant over time. Data from the roflumilast clinical programme did not provide any signals to suggest that patients would develop a tolerance or resistance to roflumilast. An analysis of time to first and subsequent exacerbations has confirmed the sustained effect of roflumilast on exacerbations [35]. Assumptions of shorter durations of reduction of the exacerbation effect in a sensitivity analysis demonstrated a stable incremental cost-effectiveness ratio.

### Reduction of hospital exacerbation rates

The pivotal roflumilast clinical trials [6] were designed to estimate the statistically significant reduction rates of community-treated exacerbations and hospital-treated exacerbations combined. However, these studies were not powered to produce a statistically significant reduction ratio of hospital-treated exacerbations. The survival benefits of roflumilast treatment in the model are partly attributed to hospital CFR and based on the assumption that exacerbation rates result in certain proportions of hospital-treated exacerbations as observed in the pivotal studies (0.155 and 0.244 in severe and very severe COPD respectively [19]). These assumptions could be further validated by including the review of available data on hospitalisations for COPD.

### Comparator regimens and relation to the UK clinical practice

The studies M2-124 and M2-125 used to populate the economic model provided a combination of evidence on the treatment effect of roflumilast on the reduction of exacerbations and improvement of lung function in patients with severe and very severe COPD treated with concomitant LABA. No direct evidence is available on the comparative effectiveness of roflumilast versus other COPD regimens representing a wide range of treatments available in the UK. An attempt was made to synthesise various studies in a network meta-analysis study in 2011 [36] that reported estimates of the relative reduction of exacerbation rates in patients treated with roflumilast versus a range of other regimens. With a wide range of comparators, the relative reduction of exacerbations had a similar magnitude to the RRR drawn from the studies M2-124 and M2-125. However, due to the nature of MTC, data in the study [36] were highly uncertain, and no effect on lung function improvement was reported.

A health economics study [37] based on the RRRs from the MTC [36] showed that in the UK setting, roflumilast as an add-on therapy to LAMA + LABA/ICS has a broadly similar exacerbation reduction ratio of 0.84 (95 % CI 0.74–0.95) and a comparable incremental cost-effectiveness of £16,566 per QALY gained.

The cost-effectiveness model in this study attempts to synthesise the best practices in COPD pharmacoeconomic modelling and uses FEV_1_-based definitions of COPD severity stages; the model is based on direct evidence derived from a pooled analysis of two pivotal RCTs. The analysis presented in this study suggests that adding roflumilast to concomitant bronchodilator therapy (LABA) can be an effective treatment option with an incremental cost-effectiveness ratio that is within a widely accepted range, i.e. £20,000-£30,000 per QALY gained.

## Abbreviations

95 % CI: 95 % confidence interval
CEAC: Cost-effectiveness acceptability curve
CFR: Case fatality rate (due to hospital-treated exacerbations)
COPD: Chronic obstructive pulmonary disease
EMA: European Medicines Agency
FEV_1 _%: Forced expiratory volume in 1 s as percentage of the predicted value
FEV_1_: Forced expiratory volume in 1 s
GP: General practitioner
HRG: Healthcare resource group
ICER: Incremental cost-effectiveness ratio
ICS: Inhaled corticosteroid
LABA: Long-acting beta agonists (bronchodilators)
LAMA: Long-acting antimuscarinic
ml: Millilitres
NHS: National health service
OWSA: One-way sensitivity analysis
PSA: Probabilistic sensitivity analysis
QALY: Quality-adjusted life year
RRR: Relative ratio of exacerbation rates
SA: Sensitivity analysis
SABA: Short-acting bronchodilators
SE: Standard error
SMR: Standardised mortality ratio
SMR_B_: Standardised mortality ratio for background mortality, which excludes hospital mortality
UK: United Kingdom
